# Comment to the article “Open partial horizontal laryngectomies: a proposal for classification by the working committee on nomenclature of the European Laryngological Society” by G. Succo et al.

**DOI:** 10.1007/s00405-014-3222-0

**Published:** 2014-08-08

**Authors:** Wierzbicka Malgorzata

**Affiliations:** Department of Otolaryngology and Laryngological Oncology, University of Medical Sciences, Przybyszewskiego Strett 49, 60-355 Poznan, Poland

I wish to make the following comment to the article *Open partial horizontal laryngectomies: a proposal for classification by the working committee on nomenclature of the European Laryngological Society* by G. Succo et al.

The presented proposal of the European Laryngeal Society working committee on nomenclature for a systemic classification of open partial horizontal laryngectomies (OPHL) is very clear, simple, univocal, easy and quick to adapt to the everyday practice in Head and Neck Departments dealing with laryngeal cancer surgery.

Nevertheless, horizontal glottectomy was not included in OPHL Type II procedures. Among the different surgical options proposed for the treatment of glottic neoplasms involving the anterior commissure (AC) this type of laryngectomy was proposed in 1978 by Calearo and Teatini [1]. This technique is based on the complete removal of the vocal folds and corresponding thyroid cartilage, with reconstruction by means of a thyrocricopexy. Although in the majority of T1b glottic cancers, open surgery has been replaced by transoral laser microlaryngoscopy (TLM), in chosen cases with AC ulceration or bicordal lesions involving the AC, horizontal glottectomy is still applied [2–4]. The modification of the horizontal glottectomy and OPHL Type IIa was presented by Wen et al. [5] and Maoxiao and Renyu [6]. This treatment option is available for T2 glottic tumors involving the AC and oncological, and functional results have confirmed the validity of this procedure [7].

In my opinion, there should be a place for partial resection of the thyroid cartilage with the glottic level in the entity of OPHL.

Yours,

Wierzbicka Malgorzata
